# Hexa­kis­(propyl­ammonium) benzene-1,2,4,5-tetra­carboxyl­ate 2,5-dicarb­oxy­benzene-1,4-carboxyl­ate tetra­hydrate

**DOI:** 10.1107/S1600536812036501

**Published:** 2012-08-31

**Authors:** Sanaz Khorasani, Manuel A. Fernandes

**Affiliations:** a Molecular Sciences Institute, School of Chemistry, University of the Witwatersrand, PO Wits 2050, Johannesburg, South Africa

## Abstract

The title organic salt, 6C_3_H_10_N^+^·C_10_H_2_O_8_^4−^·C_10_H_4_O_8_^2−^·4H_2_O, contains seven independent entities in the asymmetric unit which comprises three propyl­ammonium cations, two water mol­ecules, half a 2,5-dicarb­oxy­benzene-1,4-carboxyl­ate dianion (H_2_btc^2−^) and half a benzene-1,2,4,5-tetra­carboxyl­ate tetra­anion (btc^4−^), the latter two anions being located about centres of inversion. One of the water mol­ecules is disordered over two positions in a 0.55 (2):0.45 (2) ratio. The combination of mol­ecular ions and water mol­ecules results in an extensive and complex three-dimensional network of hydrogen bonds, the network being made up of nine unique N—H⋯O inter­actions between the ammonium cations and the anions, as well as four unique O—H⋯O inter­actions between the water mol­ecules and the anions.

## Related literature

For studies involving hydrogen-bonding inter­actions, see: Pimentel & McClellan (1960 ▶); Lemmerer (2011 ▶); Arora & Pedireddi (2003 ▶); Biradha & Zaworotko (1998 ▶). For graph-set motifs in crystal structures, see: Etter *et al.* (1990 ▶); Bernstein *et al.* (1995 ▶).
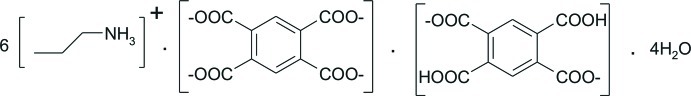


## Experimental

### 

#### Crystal data

6C_3_H_10_N^+^·C_10_H_2_O_8_^4−^·C_10_H_4_O_8_^2−^·4H_2_O
*M* *_r_* = 935.03
Triclinic, 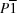

*a* = 9.9826 (2) Å
*b* = 11.0994 (2) Å
*c* = 12.4453 (2) Å
α = 107.461 (1)°
β = 90.062 (1)°
γ = 105.721 (1)°
*V* = 1261.10 (4) Å^3^
*Z* = 1
Mo *K*α radiation
μ = 0.10 mm^−1^
*T* = 173 K
0.55 × 0.33 × 0.06 mm

#### Data collection

Bruker APEXII CCD diffractometer
41269 measured reflections
6092 independent reflections
4741 reflections with *I* > 2σ(*I*)
*R*_int_ = 0.064

#### Refinement

*R*[*F* ^2^ > 2σ(*F* ^2^)] = 0.040
*wR*(*F* ^2^) = 0.118
*S* = 1.10
6092 reflections
359 parameters
36 restraints
H atoms treated by a mixture of independent and constrained refinement
Δρ_max_ = 0.40 e Å^−3^
Δρ_min_ = −0.21 e Å^−3^

### 

Data collection: *APEX2* (Bruker, 2005 ▶); cell refinement: *SAINT* (Bruker, 2005 ▶); data reduction: *SAINT*; program(s) used to solve structure: *SHELXS97* (Sheldrick, 2008 ▶); program(s) used to refine structure: *SHELXL97* (Sheldrick, 2008 ▶); molecular graphics: *PLATON* (Spek, 2009 ▶) and *SCHAKAL99* (Keller, 1999 ▶); software used to prepare material for publication: *WinGX* (Farrugia, 1999 ▶) and *PLATON*.

## Supplementary Material

Crystal structure: contains datablock(s) global, I. DOI: 10.1107/S1600536812036501/bh2430sup1.cif

Structure factors: contains datablock(s) I. DOI: 10.1107/S1600536812036501/bh2430Isup2.hkl

Additional supplementary materials:  crystallographic information; 3D view; checkCIF report

## Figures and Tables

**Table 1:** Hydrogen-bond geometry (Å, °)

*D*—H⋯*A*	*D*—H	H⋯*A*	*D*⋯*A*	*D*—H⋯*A*
N1*C*—H1*C*⋯O1*W*	0.93 (1)	1.93 (1)	2.8293 (14)	162 (1)
N1*D*—H1*F*⋯O1*B*	0.98 (1)	1.77 (1)	2.7387 (14)	172 (1)
N1*E*—H1*I*⋯O4*B*	0.95 (1)	1.87 (1)	2.8197 (13)	179 (1)
N1*C*—H1*B*⋯O1*B* ^i^	0.96 (1)	1.92 (1)	2.8598 (13)	167 (1)
N1*C*—H1*D*⋯O2*B* ^ii^	0.94 (1)	1.80 (1)	2.7269 (13)	171 (1)
N1*D*—H1*G*⋯O3*A* ^iii^	0.95 (1)	1.94 (1)	2.8725 (14)	167 (2)
N1*D*—H1*E*⋯O4*A* ^iv^	0.98 (1)	1.79 (1)	2.7642 (14)	179 (2)
N1*E*—H1*H*⋯O2*A* ^iv^	0.98 (1)	1.91 (1)	2.8493 (14)	159 (2)
N1*E*—H1*J*⋯O3*B* ^iii^	0.97 (1)	1.75 (1)	2.7170 (13)	174 (1)
O1*W*—H1*WB*⋯O3*A* ^iii^	0.880 (19)	1.94 (2)	2.8107 (14)	169.2 (17)
O1*W*—H1*WA*⋯O3*B*	0.958 (19)	1.796 (19)	2.7421 (14)	169.1 (16)
O1*A*—H1*A*⋯O2*WB* ^v^	1.00 (2)	1.54 (2)	2.513 (4)	163.1 (19)
O1*A*—H1*A*⋯O2*WA* ^v^	1.00 (2)	1.62 (2)	2.598 (5)	168.4 (19)

Symmetry codes: (i) 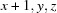; (ii) 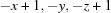; (iii) 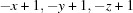; (iv) 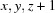; (v) 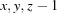.

**Fig. 1.**
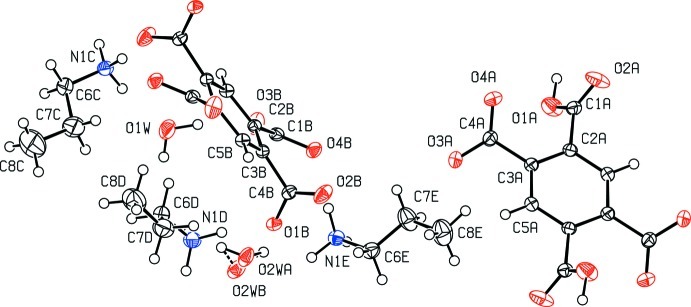
Molecules in the structure of title salt complex. Only the asymmetric unit atoms have been labeled. Displacement ellipsoids are drawn at the 50% probability level. H atoms have been drawn with spheres of arbitrary radius.

**Fig. 2.**
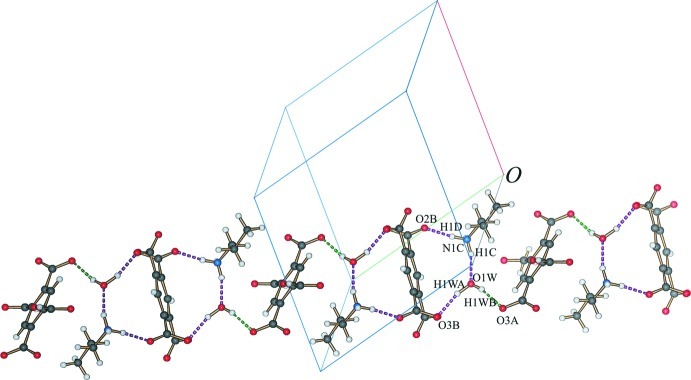
O—H···O and N—H···O hydrogen bond interactions between the water molecule O1W, the aromatic dianion (molecule A), the aromatic tetraanion (molecule B), and a propylamonium cation (molecule C).

**Fig. 3.**
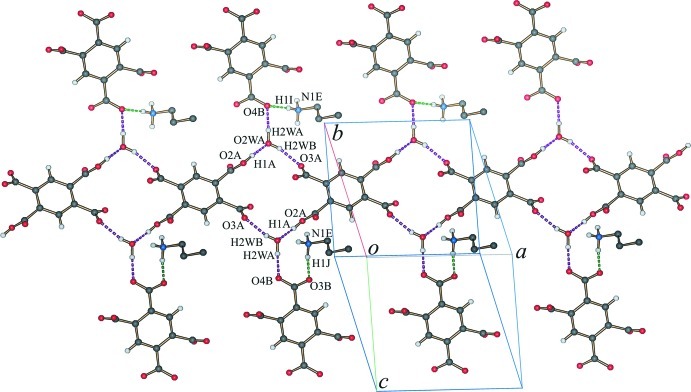
Hydrogen bond environment around the water molecule O2W. Here the water molecule hydrogen bonds to two aromatic dianions (molecule A) as both H-bond acceptor and donor. It also H-bonds to two aromatic tetra-anion molecules as H-bond donor. The combination of these interactions results in a R44 (18) ring which extends along the a axis upon translation of the unit cell.

## supplementary crystallographic information

### Comment

Intramolecular and intermolecular hydrogen bonding is of great importance in chemical and biological systems. In the crystal engineering field, hydrogen bonding plays an important role to organize molecules and assemble them to create supramolecules and control their dimensions in one-dimensional, two-dimensional, or three-dimensional networks (Lemmerer, 2011; Pimentel & McClellan, 1960; Arora & Pedireddi, 2003; Biradha & Zaworotko, 1998).

The title salt complex (Fig. 1) crystallizes in the centrosymmetric triclinic space group *P*-1 and contains seven independent entities per asymmetric unit: half a 2,5-dicarboxybenzene-1,4-carboxylate dianion (H_2_btc^2-^; molecule A), half a benzene-1,2,4,5-tetracarboxylate tetraanion (btc^4-^; molecule B), three propylammonium cations (molecules C, D and E), and two water molecules (Fig. 1). Both aromatic anions lie about inversion centres located at the centroids of the aromatic rings. One of the water molecules is disordered over two positions in a 0.55 (2):0.45 (2) ratio.

The crystal structure contains a very extensive hydrogen bonded network based on O—H···O and N—H···O interactions. Several of these involve water molecules. Water molecule O1W accepts a hydrogen from N1C located on propylammonium cation C (N1C—H1C···O1W), and donates H atoms to both aromatic anions (molecules A and B). It is therefore involved in hydrogen bonding to an ammonium cation and two aromatic anions (Fig. 2). Figure 3 shows the hydrogen bonding between the O2WA water molecule and adjacent aromatic anions. In this case the disordered water molecule only forms intermolecular hydrogen bonds with the aromatic anions as both donor and acceptor. Hydrogen bonds involving O2WA as hydrogen donor consist of O2WA—H2WA···O4B and O2WA—H2WB···O3A, and as acceptor consists of O1A—H1A···O2WA (Table 1). The combination of two O2WA water molecules and the two aromatic anions (molecules A and B) forms a hydrogen bonded ring described by the graph set *R*^4^_4_(18) (Etter *et al.*, 1990; Bernstein *et al.*, 1995). This extends as a chain of rings along the *a* axis. There are no intramolecular hydrogen bonds in this structure due to the *syn* orientation of the carboxyl hydrogen atoms. Each of the three independent propylammonium cations (molecules C, D, and E) donate three hydrogen atoms to various molecules and hence do not participate in hydrogen bond interactions with each other. Cations D and E hydrogen bond exclusively to the two aromatic anions: cation D hydrogen bonds to one B tetraanion and two A dianions, while cation E hydrogen bonds to one A dianion and two B tetraanions. The environment around propylammonium cation C is different from D and E in that it is involved in hydrogen bonding to a water molecule in addition to two B tetraanions.

### Experimental

The title organic salt was synthesized by reacting propylamine (0.27 g) with pyromellitic dianhydride (0.50 g) in the presence of THF (5 ml; not anhydrous) as a solvent, at room temperature – the presence of water resulting in ring opening of the pyromellitic dianhydride and subsequent salt formation. The solid was filtered and recrystallized in methanol, yielding colourless crystals suitable for analysis by X-ray diffraction.

### Refinement

All H atoms attached to C atoms were positioned geometrically, and allowed to ride on their parent atoms, with C—H bond lengths of 0.95 (aromatic CH), 0.99 (methylene CH_2_), or 0.98 Å (methyl CH_3_), and isotropic displacement parameters set to 1.2 (CH and CH_2_) or 1.5 times (CH_3_) the *U*_eq_ of the parent atom. Amine H atoms were placed from the difference map and refined freely. *SADI* (SAme DIstance restraint; Sheldrick, 2008) was used in the final refinements to restrain all the N—H bond lengths to reasonable values. Water H atoms were placed from the difference map and refined freely. One of the water molecules is disordered over two positions, O2WA and O2WB, in a 0.55 (2):0.45 (2) ratio.

### Crystal data

**Table:**

6C_3_H_10_N^+^·C_10_H_2_O_8_^4^^−^·C_10_H_4_O_8_^2^^−^·4H_2_O	*Z* = 1
*M**_r_* = 935.03	*F*(000) = 504
Triclinic, *P*1	*D*_x_ = 1.231 Mg m^−^^3^
Hall symbol: -P 1	Mo *K*α radiation, λ = 0.71073 Å
*a* = 9.9826 (2) Å	Cell parameters from 8121 reflections
*b* = 11.0994 (2) Å	θ = 2.2–27.2°
*c* = 12.4453 (2) Å	µ = 0.10 mm^−^^1^
α = 107.461 (1)°	*T* = 173 K
β = 90.062 (1)°	Block, colourless
γ = 105.721 (1)°	0.55 × 0.33 × 0.06 mm
*V* = 1261.10 (4) Å^3^	

### Data collection

**Table:**

Bruker APEXII CCD diffractometer	4741 reflections with *I* > 2σ(*I*)
Radiation source: fine-focus sealed tube	*R*_int_ = 0.064
Graphite monochromator	θ_max_ = 28.0°, θ_min_ = 1.7°
φ and ω scans	*h* = −13→13
41269 measured reflections	*k* = −14→14
6092 independent reflections	*l* = −16→16

### Refinement

**Table:**

Refinement on *F*^2^	Primary atom site location: structure-invariant direct methods
Least-squares matrix: full	Secondary atom site location: difference Fourier map
*R*[*F*^2^ > 2σ(*F*^2^)] = 0.040	Hydrogen site location: inferred from neighbouring sites
*wR*(*F*^2^) = 0.118	H atoms treated by a mixture of independent and constrained refinement
*S* = 1.10	*w* = 1/[σ^2^(*F*_o_^2^) + (0.0677*P*)^2^ + 0.0131*P*] where *P* = (*F*_o_^2^ + 2*F*_c_^2^)/3
6092 reflections	(Δ/σ)_max_ < 0.001
359 parameters	Δρ_max_ = 0.40 e Å^−^^3^
36 restraints	Δρ_min_ = −0.21 e Å^−^^3^
0 constraints	

### Fractional atomic coordinates and isotropic or equivalent isotropic displacement parameters (Å^2^)

**Table:**

	*x*	*y*	*z*	*U*_iso_*/*U*_eq_	Occ. (<1)
C1A	0.22708 (12)	0.52580 (12)	−0.14299 (10)	0.0232 (2)	
C2A	0.11213 (11)	0.51156 (11)	−0.06610 (9)	0.0200 (2)	
C3A	0.12378 (11)	0.47733 (11)	0.03189 (9)	0.0205 (2)	
C4A	0.25248 (12)	0.44589 (12)	0.06613 (10)	0.0244 (2)	
C5A	0.01039 (11)	0.46641 (11)	0.09698 (9)	0.0211 (2)	
H5A	0.0172	0.4434	0.1640	0.025*	
O1A	0.33910 (9)	0.62170 (11)	−0.09776 (8)	0.0409 (3)	
O2A	0.20978 (11)	0.45663 (10)	−0.24006 (8)	0.0426 (3)	
O3A	0.28839 (9)	0.48363 (9)	0.17124 (7)	0.0290 (2)	
O4A	0.31169 (11)	0.38559 (12)	−0.00919 (8)	0.0481 (3)	
H1A	0.410 (2)	0.627 (2)	−0.1536 (17)	0.073 (6)*	
C1B	0.54388 (12)	0.25468 (10)	0.46362 (9)	0.0198 (2)	
C2B	0.51859 (11)	0.12374 (10)	0.48550 (9)	0.0177 (2)	
C3B	0.38802 (11)	0.05553 (10)	0.50875 (9)	0.0179 (2)	
C4B	0.26512 (11)	0.11247 (10)	0.52874 (9)	0.0194 (2)	
C5B	0.37135 (11)	−0.06751 (10)	0.52237 (9)	0.0185 (2)	
H5B	0.2825	−0.1145	0.5374	0.022*	
O1B	0.27743 (8)	0.21010 (8)	0.61637 (6)	0.02247 (18)	
O2B	0.15747 (9)	0.05801 (9)	0.46345 (8)	0.0351 (2)	
O3B	0.66579 (9)	0.33107 (8)	0.49001 (8)	0.0317 (2)	
O4B	0.44488 (8)	0.27684 (8)	0.41868 (7)	0.02546 (19)	
C6C	1.00660 (13)	0.14661 (13)	0.78897 (10)	0.0302 (3)	
H6A	1.0776	0.2198	0.8437	0.036*	
H6B	1.0366	0.0657	0.7751	0.036*	
C7C	0.86828 (17)	0.1274 (2)	0.83874 (14)	0.0518 (4)	
H7A	0.7957	0.0592	0.7818	0.062*	
H7B	0.8419	0.2107	0.8583	0.062*	
C8C	0.8738 (3)	0.0858 (3)	0.94401 (18)	0.0825 (7)	
H8A	0.8858	−0.0028	0.9229	0.124*	
H8B	0.7865	0.0856	0.9800	0.124*	
H8C	0.9526	0.1479	0.9970	0.124*	
N1C	0.99833 (11)	0.17678 (10)	0.68130 (9)	0.0237 (2)	
H1B	1.0887 (13)	0.1969 (15)	0.6545 (12)	0.041 (4)*	
H1C	0.9581 (15)	0.2445 (13)	0.6863 (13)	0.039 (4)*	
H1D	0.9397 (15)	0.1009 (13)	0.6289 (11)	0.042 (4)*	
C6D	0.51159 (14)	0.19783 (14)	0.81853 (11)	0.0342 (3)	
H6C	0.5811	0.2313	0.8851	0.041*	
H6D	0.5588	0.1649	0.7506	0.041*	
C7D	0.39438 (16)	0.08625 (15)	0.83184 (13)	0.0429 (4)	
H7C	0.3219	0.0568	0.7678	0.051*	
H7D	0.3511	0.1179	0.9024	0.051*	
C8D	0.4457 (2)	−0.02982 (18)	0.83571 (15)	0.0575 (5)	
H8D	0.4885	−0.0615	0.7658	0.086*	
H8E	0.3666	−0.1009	0.8431	0.086*	
H8F	0.5151	−0.0017	0.9007	0.086*	
N1D	0.45926 (11)	0.30762 (11)	0.80741 (9)	0.0308 (2)	
H1G	0.5358 (15)	0.3796 (14)	0.8062 (13)	0.044 (4)*	
H1E	0.4074 (17)	0.3349 (17)	0.8726 (12)	0.056 (5)*	
H1F	0.4011 (15)	0.2756 (15)	0.7358 (10)	0.041 (4)*	
C6E	0.11925 (13)	0.35625 (13)	0.48083 (10)	0.0287 (3)	
H6E	0.0610	0.4160	0.5112	0.034*	
H6F	0.0832	0.2764	0.5041	0.034*	
C7E	0.10636 (16)	0.31785 (16)	0.35439 (12)	0.0434 (4)	
H7E	0.1654	0.2589	0.3237	0.052*	
H7F	0.1405	0.3977	0.3308	0.052*	
C8E	−0.04440 (17)	0.24798 (18)	0.30615 (14)	0.0538 (4)	
H8G	−0.0790	0.1700	0.3308	0.081*	
H8H	−0.0493	0.2210	0.2234	0.081*	
H8I	−0.1020	0.3079	0.3332	0.081*	
N1E	0.26635 (11)	0.42296 (10)	0.53008 (9)	0.0240 (2)	
H1H	0.2706 (18)	0.4358 (17)	0.6115 (10)	0.054 (5)*	
H1I	0.3266 (14)	0.3730 (14)	0.4935 (12)	0.038 (4)*	
H1J	0.2960 (15)	0.5099 (11)	0.5212 (11)	0.032 (4)*	
O1W	0.82657 (12)	0.33934 (11)	0.67230 (10)	0.0453 (3)	
H1WA	0.7720 (18)	0.3259 (17)	0.6042 (15)	0.055 (5)*	
H1WB	0.7964 (18)	0.3927 (19)	0.7282 (15)	0.056 (5)*	
O2WA	0.5131 (5)	0.6023 (11)	0.7471 (7)	0.0361 (15)	0.55 (2)
O2WB	0.5446 (9)	0.6686 (15)	0.7894 (10)	0.042 (2)	0.45 (2)
H2WA	0.534 (2)	0.662 (2)	0.7082 (18)	0.070 (6)*	
H2WB	0.589 (2)	0.606 (2)	0.7792 (17)	0.056 (5)*	

### Atomic displacement parameters (Å^2^)

**Table:**

	*U*^11^	*U*^22^	*U*^33^	*U*^12^	*U*^13^	*U*^23^
C1A	0.0222 (6)	0.0280 (6)	0.0230 (6)	0.0108 (5)	0.0057 (4)	0.0098 (5)
C2A	0.0189 (5)	0.0224 (5)	0.0172 (5)	0.0067 (4)	0.0027 (4)	0.0035 (4)
C3A	0.0192 (5)	0.0227 (6)	0.0185 (5)	0.0071 (4)	0.0008 (4)	0.0038 (4)
C4A	0.0205 (5)	0.0312 (6)	0.0247 (6)	0.0097 (5)	0.0031 (4)	0.0113 (5)
C5A	0.0215 (5)	0.0247 (6)	0.0173 (5)	0.0075 (4)	0.0013 (4)	0.0061 (4)
O1A	0.0236 (5)	0.0557 (6)	0.0325 (5)	−0.0013 (4)	0.0075 (4)	0.0093 (5)
O2A	0.0454 (6)	0.0454 (6)	0.0258 (5)	0.0064 (5)	0.0159 (4)	0.0005 (4)
O3A	0.0256 (4)	0.0408 (5)	0.0241 (4)	0.0149 (4)	−0.0009 (3)	0.0104 (4)
O4A	0.0478 (6)	0.0829 (8)	0.0320 (5)	0.0482 (6)	0.0138 (4)	0.0180 (5)
C1B	0.0254 (6)	0.0176 (5)	0.0188 (5)	0.0095 (4)	0.0049 (4)	0.0062 (4)
C2B	0.0209 (5)	0.0161 (5)	0.0169 (5)	0.0069 (4)	0.0021 (4)	0.0045 (4)
C3B	0.0202 (5)	0.0168 (5)	0.0168 (5)	0.0073 (4)	0.0005 (4)	0.0036 (4)
C4B	0.0190 (5)	0.0168 (5)	0.0247 (5)	0.0061 (4)	0.0040 (4)	0.0088 (4)
C5B	0.0186 (5)	0.0168 (5)	0.0199 (5)	0.0046 (4)	0.0036 (4)	0.0059 (4)
O1B	0.0239 (4)	0.0215 (4)	0.0227 (4)	0.0110 (3)	0.0034 (3)	0.0039 (3)
O2B	0.0233 (4)	0.0268 (5)	0.0467 (5)	0.0094 (4)	−0.0095 (4)	−0.0029 (4)
O3B	0.0302 (5)	0.0196 (4)	0.0453 (5)	0.0012 (4)	−0.0054 (4)	0.0156 (4)
O4B	0.0277 (4)	0.0292 (4)	0.0293 (4)	0.0152 (4)	0.0071 (3)	0.0169 (4)
C6C	0.0317 (7)	0.0317 (7)	0.0294 (6)	0.0119 (5)	0.0014 (5)	0.0103 (5)
C7C	0.0448 (9)	0.0760 (12)	0.0487 (9)	0.0236 (8)	0.0202 (7)	0.0341 (9)
C8C	0.0876 (15)	0.121 (2)	0.0633 (13)	0.0335 (14)	0.0300 (11)	0.0605 (14)
N1C	0.0218 (5)	0.0231 (5)	0.0249 (5)	0.0074 (4)	0.0028 (4)	0.0044 (4)
C6D	0.0303 (7)	0.0449 (8)	0.0270 (6)	0.0162 (6)	0.0000 (5)	0.0059 (6)
C7D	0.0480 (9)	0.0474 (9)	0.0357 (7)	0.0175 (7)	0.0105 (6)	0.0130 (7)
C8D	0.0827 (13)	0.0550 (10)	0.0442 (9)	0.0288 (9)	0.0124 (9)	0.0212 (8)
N1D	0.0248 (5)	0.0359 (6)	0.0268 (6)	0.0090 (5)	0.0018 (4)	0.0024 (5)
C6E	0.0285 (6)	0.0297 (6)	0.0328 (7)	0.0115 (5)	0.0082 (5)	0.0140 (5)
C7E	0.0422 (8)	0.0494 (9)	0.0337 (7)	−0.0008 (7)	0.0014 (6)	0.0182 (7)
C8E	0.0459 (9)	0.0584 (10)	0.0482 (9)	0.0011 (8)	−0.0083 (7)	0.0161 (8)
N1E	0.0296 (5)	0.0201 (5)	0.0264 (5)	0.0104 (4)	0.0062 (4)	0.0100 (4)
O1W	0.0521 (6)	0.0462 (6)	0.0359 (6)	0.0330 (5)	−0.0143 (5)	−0.0066 (5)
O2WA	0.0229 (13)	0.063 (4)	0.042 (2)	0.0199 (17)	0.0125 (14)	0.038 (3)
O2WB	0.032 (2)	0.072 (5)	0.048 (3)	0.030 (3)	0.020 (2)	0.042 (4)

### Geometric parameters (Å, º)

**Table:**

C1A—O2A	1.2041 (14)	N1C—H1C	0.930 (12)
C1A—O1A	1.3017 (15)	N1C—H1D	0.938 (11)
C1A—C2A	1.5007 (15)	C6D—N1D	1.4910 (18)
C2A—C5A^i^	1.3859 (15)	C6D—C7D	1.506 (2)
C2A—C3A	1.3953 (15)	C6D—H6C	0.9900
C3A—C5A	1.3923 (15)	C6D—H6D	0.9900
C3A—C4A	1.5117 (16)	C7D—C8D	1.523 (2)
C4A—O4A	1.2309 (15)	C7D—H7C	0.9900
C4A—O3A	1.2665 (14)	C7D—H7D	0.9900
C5A—C2A^i^	1.3859 (15)	C8D—H8D	0.9800
C5A—H5A	0.9500	C8D—H8E	0.9800
O1A—H1A	1.00 (2)	C8D—H8F	0.9800
C1B—O4B	1.2504 (13)	N1D—H1G	0.948 (12)
C1B—O3B	1.2572 (14)	N1D—H1E	0.978 (12)
C1B—C2B	1.5128 (15)	N1D—H1F	0.977 (11)
C2B—C5B^ii^	1.3933 (15)	C6E—N1E	1.4889 (16)
C2B—C3B	1.3976 (15)	C6E—C7E	1.4973 (18)
C3B—C5B	1.3920 (15)	C6E—H6E	0.9900
C3B—C4B	1.5142 (14)	C6E—H6F	0.9900
C4B—O2B	1.2376 (14)	C7E—C8E	1.521 (2)
C4B—O1B	1.2638 (13)	C7E—H7E	0.9900
C5B—C2B^ii^	1.3933 (15)	C7E—H7F	0.9900
C5B—H5B	0.9500	C8E—H8G	0.9800
C6C—N1C	1.4827 (15)	C8E—H8H	0.9800
C6C—C7C	1.5024 (19)	C8E—H8I	0.9800
C6C—H6A	0.9900	N1E—H1H	0.979 (12)
C6C—H6B	0.9900	N1E—H1I	0.952 (11)
C7C—C8C	1.519 (2)	N1E—H1J	0.970 (11)
C7C—H7A	0.9900	O1W—H1WA	0.958 (19)
C7C—H7B	0.9900	O1W—H1WB	0.880 (19)
C8C—H8A	0.9800	O2WA—H2WA	0.91 (2)
C8C—H8B	0.9800	O2WA—H2WB	0.84 (2)
C8C—H8C	0.9800	O2WB—H2WA	1.00 (2)
N1C—H1B	0.955 (12)	O2WB—H2WB	0.89 (2)
			
O2A—C1A—O1A	124.64 (11)	H1B—N1C—H1D	109.4 (13)
O2A—C1A—C2A	120.64 (11)	H1C—N1C—H1D	106.6 (13)
O1A—C1A—C2A	114.60 (10)	N1D—C6D—C7D	111.46 (11)
C5A^i^—C2A—C3A	120.07 (10)	N1D—C6D—H6C	109.3
C5A^i^—C2A—C1A	116.65 (10)	C7D—C6D—H6C	109.3
C3A—C2A—C1A	123.27 (10)	N1D—C6D—H6D	109.3
C5A—C3A—C2A	118.52 (10)	C7D—C6D—H6D	109.3
C5A—C3A—C4A	119.36 (10)	H6C—C6D—H6D	108.0
C2A—C3A—C4A	122.06 (10)	C6D—C7D—C8D	111.73 (13)
O4A—C4A—O3A	126.02 (11)	C6D—C7D—H7C	109.3
O4A—C4A—C3A	117.91 (10)	C8D—C7D—H7C	109.3
O3A—C4A—C3A	116.06 (10)	C6D—C7D—H7D	109.3
C2A^i^—C5A—C3A	121.40 (10)	C8D—C7D—H7D	109.3
C2A^i^—C5A—H5A	119.3	H7C—C7D—H7D	107.9
C3A—C5A—H5A	119.3	C7D—C8D—H8D	109.5
C1A—O1A—H1A	110.4 (11)	C7D—C8D—H8E	109.5
O4B—C1B—O3B	125.49 (10)	H8D—C8D—H8E	109.5
O4B—C1B—C2B	118.29 (10)	C7D—C8D—H8F	109.5
O3B—C1B—C2B	116.19 (9)	H8D—C8D—H8F	109.5
C5B^ii^—C2B—C3B	119.25 (10)	H8E—C8D—H8F	109.5
C5B^ii^—C2B—C1B	118.35 (9)	C6D—N1D—H1G	109.7 (10)
C3B—C2B—C1B	122.35 (10)	C6D—N1D—H1E	108.3 (11)
C5B—C3B—C2B	118.92 (10)	H1G—N1D—H1E	109.5 (14)
C5B—C3B—C4B	117.47 (9)	C6D—N1D—H1F	107.8 (9)
C2B—C3B—C4B	123.45 (9)	H1G—N1D—H1F	109.1 (13)
O2B—C4B—O1B	123.98 (10)	H1E—N1D—H1F	112.5 (14)
O2B—C4B—C3B	119.26 (10)	N1E—C6E—C7E	112.22 (10)
O1B—C4B—C3B	116.65 (9)	N1E—C6E—H6E	109.2
C3B—C5B—C2B^ii^	121.83 (10)	C7E—C6E—H6E	109.2
C3B—C5B—H5B	119.1	N1E—C6E—H6F	109.2
C2B^ii^—C5B—H5B	119.1	C7E—C6E—H6F	109.2
N1C—C6C—C7C	111.51 (11)	H6E—C6E—H6F	107.9
N1C—C6C—H6A	109.3	C6E—C7E—C8E	111.17 (13)
C7C—C6C—H6A	109.3	C6E—C7E—H7E	109.4
N1C—C6C—H6B	109.3	C8E—C7E—H7E	109.4
C7C—C6C—H6B	109.3	C6E—C7E—H7F	109.4
H6A—C6C—H6B	108.0	C8E—C7E—H7F	109.4
C6C—C7C—C8C	111.48 (15)	H7E—C7E—H7F	108.0
C6C—C7C—H7A	109.3	C7E—C8E—H8G	109.5
C8C—C7C—H7A	109.3	C7E—C8E—H8H	109.5
C6C—C7C—H7B	109.3	H8G—C8E—H8H	109.5
C8C—C7C—H7B	109.3	C7E—C8E—H8I	109.5
H7A—C7C—H7B	108.0	H8G—C8E—H8I	109.5
C7C—C8C—H8A	109.5	H8H—C8E—H8I	109.5
C7C—C8C—H8B	109.5	C6E—N1E—H1H	108.8 (10)
H8A—C8C—H8B	109.5	C6E—N1E—H1I	110.5 (9)
C7C—C8C—H8C	109.5	H1H—N1E—H1I	111.7 (13)
H8A—C8C—H8C	109.5	C6E—N1E—H1J	109.6 (9)
H8B—C8C—H8C	109.5	H1H—N1E—H1J	106.8 (13)
C6C—N1C—H1B	110.5 (9)	H1I—N1E—H1J	109.4 (12)
C6C—N1C—H1C	113.5 (9)	H1WA—O1W—H1WB	107.7 (15)
H1B—N1C—H1C	110.0 (13)	H2WA—O2WA—H2WB	106.7 (19)
C6C—N1C—H1D	106.7 (9)	H2WA—O2WB—H2WB	96 (2)
			
O2A—C1A—C2A—C5A^i^	−60.87 (16)	O4B—C1B—C2B—C3B	−30.62 (15)
O1A—C1A—C2A—C5A^i^	115.26 (12)	O3B—C1B—C2B—C3B	151.27 (11)
O2A—C1A—C2A—C3A	118.78 (14)	C5B^ii^—C2B—C3B—C5B	−0.60 (17)
O1A—C1A—C2A—C3A	−65.09 (15)	C1B—C2B—C3B—C5B	176.82 (9)
C5A^i^—C2A—C3A—C5A	−0.15 (18)	C5B^ii^—C2B—C3B—C4B	174.69 (9)
C1A—C2A—C3A—C5A	−179.80 (10)	C1B—C2B—C3B—C4B	−7.88 (16)
C5A^i^—C2A—C3A—C4A	176.98 (10)	C5B—C3B—C4B—O2B	−66.68 (14)
C1A—C2A—C3A—C4A	−2.66 (17)	C2B—C3B—C4B—O2B	117.96 (12)
C5A—C3A—C4A—O4A	140.12 (12)	C5B—C3B—C4B—O1B	109.48 (11)
C2A—C3A—C4A—O4A	−36.99 (17)	C2B—C3B—C4B—O1B	−65.87 (14)
C5A—C3A—C4A—O3A	−39.17 (16)	C2B—C3B—C5B—C2B^ii^	0.62 (17)
C2A—C3A—C4A—O3A	143.72 (11)	C4B—C3B—C5B—C2B^ii^	−174.96 (9)
C2A—C3A—C5A—C2A^i^	0.16 (18)	N1C—C6C—C7C—C8C	175.47 (16)
C4A—C3A—C5A—C2A^i^	−177.05 (10)	N1D—C6D—C7D—C8D	176.37 (11)
O4B—C1B—C2B—C5B^ii^	146.82 (10)	N1E—C6E—C7E—C8E	179.07 (13)
O3B—C1B—C2B—C5B^ii^	−31.29 (14)		

Symmetry codes: (i) −*x*, −*y*+1, −*z*; (ii) −*x*+1, −*y*, −*z*+1.

### Hydrogen-bond geometry (Å, º)

**Table:**

*D*—H···*A*	*D*—H	H···*A*	*D*···*A*	*D*—H···*A*
N1*C*—H1*C*···O1*W*	0.93 (1)	1.93 (1)	2.8293 (14)	162 (1)
N1*D*—H1*F*···O1*B*	0.98 (1)	1.77 (1)	2.7387 (14)	172 (1)
N1*E*—H1*I*···O4*B*	0.95 (1)	1.87 (1)	2.8197 (13)	179 (1)
N1*C*—H1*B*···O1*B*^iii^	0.96 (1)	1.92 (1)	2.8598 (13)	167 (1)
N1*C*—H1*D*···O2*B*^ii^	0.94 (1)	1.80 (1)	2.7269 (13)	171 (1)
N1*D*—H1*G*···O3*A*^iv^	0.95 (1)	1.94 (1)	2.8725 (14)	167 (2)
N1*D*—H1*E*···O4*A*^v^	0.98 (1)	1.79 (1)	2.7642 (14)	179 (2)
N1*E*—H1*H*···O2*A*^v^	0.98 (1)	1.91 (1)	2.8493 (14)	159 (2)
N1*E*—H1*J*···O3*B*^iv^	0.97 (1)	1.75 (1)	2.7170 (13)	174 (1)
O1*W*—H1*WB*···O3*A*^iv^	0.880 (19)	1.94 (2)	2.8107 (14)	169.2 (17)
O1*W*—H1*WA*···O3*B*	0.958 (19)	1.796 (19)	2.7421 (14)	169.1 (16)
O1*A*—H1*A*···O2*WB*^vi^	1.00 (2)	1.54 (2)	2.513 (4)	163.1 (19)
O1*A*—H1*A*···O2*WA*^vi^	1.00 (2)	1.62 (2)	2.598 (5)	168.4 (19)

Symmetry codes: (ii) −*x*+1, −*y*, −*z*+1; (iii) *x*+1, *y*, *z*; (iv) −*x*+1, −*y*+1, −*z*+1; (v) *x*, *y*, *z*+1; (vi) *x*, *y*, *z*−1.

## References

[bb1] Arora, K. K. & Pedireddi, V. R. (2003). *J. Org. Chem.* **68**, 9177–9185. 10.1021/jo034434z 14629133

[bb2] Bernstein, J., Davis, R. E., Shimoni, L. & Chang, N.-L. (1995). *Angew. Chem. Int. Ed. Engl.* **34**, 1555–1573.

[bb3] Biradha, K. & Zaworotko, M. J. (1998). *Cryst. Eng.* **1**, 67–78.

[bb4] Bruker (2005). *APEX2* and *SAINT* Bruker AXS Inc., Madison, Wisconsin, USA.

[bb5] Etter, M. C., MacDonald, J. C. & Bernstein, J. (1990). *Acta Cryst.* B**46**, 256–262. 10.1107/s0108768189012929 2344397

[bb6] Farrugia, L. J. (1999). *J. Appl. Cryst.* **32**, 837–838.

[bb7] Keller, E. (1999). *SCHAKAL99* University of Freiberg, Germany.

[bb8] Lemmerer, A. (2011). *Cryst. Growth Des.* **11**, 583–593.

[bb9] Pimentel, G. C. & McClellan, A. L. (1960). In *The Hydrogen Bond* San Francisco: Freeman.

[bb10] Sheldrick, G. M. (2008). *Acta Cryst.* A**64**, 112–122. 10.1107/S0108767307043930 18156677

[bb11] Spek, A. L. (2009). *Acta Cryst.* D**65**, 148–155. 10.1107/S090744490804362X PMC2631630 19171970

